# Anti-Inflammatory Activity and Quality Control of *Erysimum cheiri* (L.) Crantz

**DOI:** 10.1155/2021/5526644

**Published:** 2021-06-08

**Authors:** Ghazaleh Mosleh, Amir Azadi, Sedigheh Khademian, Reza Heidari, Abdolali Mohagheghzadeh

**Affiliations:** ^1^Phytopharmaceutical Technology and Traditional Medicine Incubator, Shiraz University of Medical Sciences, Shiraz, Iran; ^2^Department of Pharmaceutics, Faculty of Pharmacy, Shiraz University of Medical Sciences, Shiraz, Iran; ^3^Department of Phytopharmaceuticals (Traditional Pharmacy), Faculty of Pharmacy, Shiraz University of Medical Sciences, Shiraz, Iran; ^4^Pharmaceutical Sciences Research Center, Shiraz University of Medical Sciences, Shiraz, Iran

## Abstract

Wallflower (*Erysimum cheiri*) is employed as a popular herbal drug in traditional Persian medicine. Topical formulations including cerates, lotions, sitz baths, and poultices for inflammatory disorders such as arthritis, anal fissure, endometriosis, and mastitis are known. However, there is no monograph in current pharmacopoeia for the wallflower drug. The present study is aimed to screen *in vitro* anti-inflammatory activity of wallflower and perform quality control and characterization tests for different organs of the herb. In this regard, albumin denaturation activity, macroscopic and microscopic, phytochemical, HPTLC, and FT-IR characteristics were investigated. Wallflower showed strong anti-inflammatory activity compared to diclofenac sodium. The root (1.25, 2.5, and 5 mg/mL) and flower (10 mg/mL) extract exhibited higher anti-inflammatory activities than that of other plant organs at the same concentrations. Moreover, total ash was found higher in aerial parts (21.52 ± 0.06%) than flower (11.01 ± 0.03%), root (5.03 ± 0.03%), and seed (6.95 ± 0.06%), while water-soluble ash was higher in seed (34.89 ± 0.26%) than flower (5.00 ± 0.03%), aerial parts (7.16 ± 0.06%), and root (5.04 ± 0.01%). Acid-insoluble ash and sulphated ash were higher in root (9.50 ± 0.04%) and aerial part (28.37 ± 0.57%), respectively. In addition, loss on drying was ranged from 2.20 ± 0.20% in flowers to 6.00 ± 0.10% in aerial parts. On the other hand, HPTLC analysis verified cardenolide compounds in all organs of the herb, and quercetin was detected in the flavonoid fingerprint of acid hydrolysed flowers. According to FT-IR results, the observed spectral region at ~3500 cm^−1^ attributed to -OH stretching vibration. Also, C–H (~2900-2950 cm^−1^), isothiocyanate (~2340 cm^−1^), -C=O (~1740 cm^−1^), conjugated C=C of the aromatic ring (~1650 cm^−1^), and structure of the aromatic group (~1200-1000 cm^−1^) were monitored. This work is the first study to the best of our knowledge, suggesting wallflower as a potential drug candidate with the basis for a monograph in addition to initial *in vitro* anti-inflammatory data.

## 1. Introduction

Wallflower (*Erysimum cheiri* (L.) Crantz, synonyms: *Cheiranthus cheiri* L.) from the Brassicaceae family is used as a popular drug in many traditional medicine systems since ancient times [1, 2]. In medieval Persia, the wallflower was called “*Cheiri*,” which was further adopted by Linnaeus to make the scientific name of “*Cheiranthus cheiri*” for this drug. “*Cheiranthus*” is made up of two smaller words, “*cheir*” and “*anthos*”, which “*cheir*” originates from its medieval Persian name, and “*anthos*” (*άνθος*) is a Latin word meaning flower [3]. Moreover, it is considered that the phrase “*Cheiri*” may pertain to “*Cheiros*” (*χειρός*), meaning a man's hand in Latin because the flower was used as a bouquet during ancient Greek festivals [3, 4]. The medical indications of the wallflower are well documented in traditional manuscripts and folk medicines around the world [1, 5, 6]. Traditional wallflower formulations prepared in various dosage forms, including cerates, lotions, sitz baths, and poultices, have frequently been recommended for acute and chronic inflammatory conditions such as arthritis, endometriosis, and mastitis in Persian medicine [1, 7–9].

Fingerprinting is a quality control technique accepted by the World Health Organization (WHO) to address the comprehensive nature of medicinal herbs [10]. Phytochemical studies on wallflower revealed several cardiotonic steroids such as cheiroside A, cheirotoxin, and neouzarin in the seeds [1]. Moreover, a thin-layer chromatography (TLC) study on the seeds of a wallflower, represented more than 30 cardiac glycoside zones having a remarkable similarity with the TLC fingerprint of *Cheiranthus × allionii* Bois [11]. On the other hand, the presence of several glucosinolates and isothiocyanates such as cheirolin and iberin have been verified in wallflower seed, while cheirolin was detected in the essential oil of flower, leaf, and fruit. Also, some flavonoids such as isorhamnetin, kaempferol, and quercetin derivatives were detected in wallflower herb [1, 12].

Although various medical properties have been described for wallflower in traditional medicine, there is no monograph in the current pharmacopeia, and the phytochemical composition and potential effects of the herb are largely unknown in modern medicine. There are only a few investigations conducted on the pharmacological activities of the wallflower. The topical indication of wallflower extract on murine skin demonstrated chemopreventive effects [13]. Furthermore, the ethanolic extract of wallflower exhibited toxicity effects on Calf kidney cells and dose-dependent antiviral properties in previous *in vitro* studies [14]. Another investigation revealed the antibacterial and antifungi properties of the herb [15]. Also, cardiac glycoside extract of wallflower has shown digitalin-like properties [16]. According to our previous published clinical study on acute anal fissure, the topical application of a traditional Persian formulation containing wallflower showed comparable effects to diltiazem 2% gel [9]. Considering the large variety of cardiac glycosides in wallflower seed, especially the high content of 11-*α*-hydroxycardenolides, a former study has predicted several new pharmacotherapeutic properties in addition to the cardiotonic effects for this seed [17].

Traditional medicine is a rich source for discovering novel therapeutic agents. Expansion of using traditional drugs has emerged to identify and standardization of applied herbs in such formulations [9, 18]. A literature survey revealed no previous attempts for evaluating the anti-inflammatory properties of wallflower extracts. In addition, no studies were found on fingerprinting of different organs of wallflower except seeds. This study aimed to provide information on the anti-inflammatory activity and diagnostic characteristics of different organs of the wallflower herb. The results of the present study could be useful in establishing a pharmacopoeial monograph to be served as a reference for accurate identification and authentication of *E. cheiri*.

## 2. Materials and Methods

### 2.1. Plant Materials and Chemicals

Wallflower was collected from Shiraz city. It was identified as *Erysimum cheiri* (L.) Crantz [19] by an expert botanist in Shiraz Traditional Pharmacy Department, School of Pharmacy, and a sample was deposited in the Herbarium Centre of School of Pharmacy, Shiraz University of Medical Sciences, under the voucher number of 784. On the other hand, all chemicals, unless otherwise stated, were obtained from Sigma–Aldrich chemicals, and all the applied solvents were of analytical grade.

### 2.2. *In Vitro* Anti-Inflammatory Activity (Protein Denaturation Inhibitory Activity)

#### 2.2.1. Preparation of Herbal Extracts

In order to prepare extracts, the method was done as described by Sharifi-Rad et al. (2015) with some modifications. Ten g of air-dried powder of each sample was dissolved in ethanol 85% at room temperature for 48 hours. Then, it was vacuum filtered, and the filtrate was concentrated to remove the residual solvent [20]. The extract yields (*w/w* %) of flowers, aerial parts, roots, and seeds were calculated as 28.8%, 13.8%, 6.2%, and 13.36%, respectively. The obtained extracts were stored at 4°C until the assays were carried out.

#### 2.2.2. The Albumin Denaturation Inhibitory Activity

The anti-inflammatory activity was investigated through the method as described by Sharifi-Rad et al. (2015). Briefly, 1 mL of various concentrations (1.25, 2.5, 5, 10 mg/mL in distilled water) of herbal extracts, including flower, aerial part, root, and seed, was blended with 2 mL of egg albumin and 3 mL of phosphate-buffered saline (PBS, pH 6.5). The mixtures were incubated at 25°C for 15 min. Then, they were heated for 12 min at 65°C, and after cooling, the absorbance was recorded via Epoch spectrophotometer system (Biotek instruments) at 660 nm. Diclofenac sodium (Padtan Teb Co., Iran) was used as the standard drug in the same amounts as wallflower extracts. The percentage inhibition of albumin denaturation was estimated using the following formula [20, 21].

(1) $$\begin{array}{c} \%\text{Inhibition}=\frac{\text{Absorbance of test sample}-\text{absorbance of control}}{\text{Absorbance of control}}\times 100. \end{array}$$

#### 2.2.3. Statistical Analysis

The data obtained from the albumin denaturation inhibitory test are presented as mean ± SEM (*n* = 3). Data were analyzed by one-way analysis of variance (ANOVA) with Tukey as the *post hoc* test. Values with *p* < 0.05 were considered as statistically significant differences between groups.

### 2.3. Macroscopic and Microscopic Evaluations

**Figure 1:**
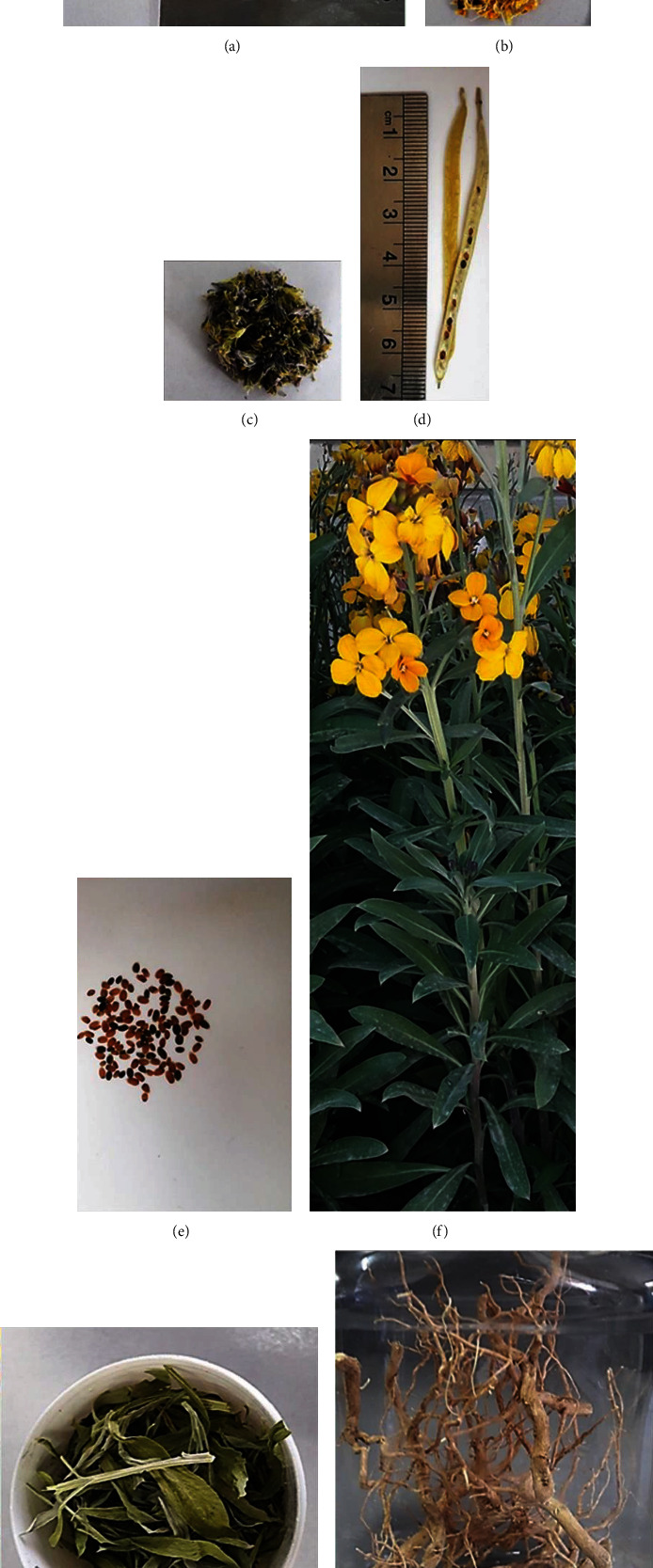
*Erysimum cheiri*: (a) flower, (b) petal, (c) sepal, (d) fruit, (e) seed, (f) fresh plant, (g) aerial part, and (h) root.

Flowers, leaves, stems, fruits, roots, and wallflower seeds were studied (Figure 1). In order to microscopic characterization, sections were cut manually and were simple and double-stained by methylene blue (0.5 mg/mL) and Congo red (10 mg/mL). Then, sections were bleached with sodium hypochlorite and fixed with acetic acid 1%. Finally, the slides were observed, and photomicrographs were captured using the optical Canon microscope attached to a Cannon camera [22].

### 2.4. Determination of Ash Value and Loss on Drying

Total ash content, acid-insoluble ash, water soluble ash, sulphated ash, and loss on drying values of different organs of wallflower were determined as previously described [23–25].

### 2.5. High-Performance Thin Layer Chromatography (HPTLC)

HPTLC analysis was performed to get the fingerprints of flavonoids and cardenolides in different organs of the wallflower. The process was carried out on a CAMAG HPTLC system equipped with an automatic TLC sampler ATS4, TLC scanner 3, and an integrated software Win-CATS was used for the study. Precoated silica gel 60F254 aluminum plates (0.2 mm thick) were used, and the method was done according to Bladt and Wagner, 2009 [12].

#### 2.5.1. HPTLC Fingerprint of Flavonoids

*(1) Preparation of Extracts from Plant Materials*. The methanol extract was obtained by adding 10 mL methanol to 1 g powder of dry plant material and keeping it for 5 min in water-bath 60°C [12]. On the other hand, in order to prepare the acid hydrolysed sample, 1 g of air-dried powder was extracted with 50 mL methanol, and it was heated for 30 min at a water bath. Two mL of this solution was dissolved in 2 mL of HCl (1.1 M) : MeOH (60 : 40 *v*/*v*) mixture and heated in a water bath for 30 min, then dried in a desiccator, dissolved in 2 mL methanol, and filtered [26].

*(2) Preparation of Standard Solution*. The amount of 0.1 mg quercetin was dissolved in methanol (2 mL) in a volumetric flask. It was used as a working standard solution for the analysis.

*(3) Preparation of Natural Products-Polyethylene Glycol Reagent (NP/PEG)*. The plate was reacted with methanolic diphenylboric acid-*p*-ethylamino ester 1% (= diphenylboryloxyethylamine, Natural Product reagent), followed by ethanolic polyethylene glycol-4000 (PEG) 5% (10 mL and 8 mL, respectively).

*(4) Method Development*. The solvent system of n-hexane : EtAcOH : AcOH (5 : 3 : 1) was examined in this study. Ten *μ*L of each solution was applied as bands on a 10 × 20 cm TLC silica gel 60 F_254_ plates. Application positions were 10 mm from the sides and 10 mm from the bottom of the plates. Mobile phase components were mixed before use, and the development chamber was saturated with mobile phase vapor for 10 min before each run. Ascending development of the plate, migration distance 80 mm, was performed at 25°C. After development, the TLC plate was dried, and then, the Natural Product reagent was sprayed on the plate. Densitometric scanning was performed with absorbance mode at 366 nm with winCATS software, using the deuterium light source. The scanning speed was at 20 mm/s and the data resolution at 100 m/step [12].

#### 2.5.2. HPTLC Fingerprint of Cardenolides

*(1) Preparation of Cardenolide Extracts*. To prepare cardenolide extracts, 30 mL ethanol 50% and 10 mL lead acetate 10% were added to 2 g of air-dried powders, and the mixtures were heated for 15 min under reflux. After cooling and filtration, the solution was extracted with shaking gently in a decanter 3 times with 15 mL dichloromethane : isopropanol (3 : 2). The combined lower phases were filtered over anhydrous sodium sulphate to be dehydrated and evaporated to dryness. The residue was dissolved in 1 mL dichloromethane : isopropanol (3 : 2), and it was adopted for HPTLC [12].

*(2) Preparation of Standard Solution*. One mg digoxin (Zahravi Pharmaceutical Co., Tabriz) was accurately weighed into a 10 mL volumetric flask and dissolved in dichloromethane : isopropanol (3 : 2) up to 10 mL.

*(3) Preparation of Kedde Reagent*. Five mL of freshly prepared ethanolic 3,5-dinitrobenzoic acid 3% was mixed with 5 mL NaOH 2 M. Kedde reagent was prepared freshly before the test.

*(4) Method Development*. The solvent system was EtAcOH : MeOH : H_2_O (81 : 11 : 8). Twenty-five *μ*L of each solution was applied as bands on a 10 × 20 cm TLC plate. Application positions were 10 mm from the sides and 10 mm from the bottom of the plates. Mobile phase components were mixed before use, and the development chamber was saturated with mobile phase vapor for 10 min before each run. Ascending development of the plate, migration distance 80 mm, was performed at 25°C. After development, the TLC plate was dried, and then, the Kedde reagent was sprayed on the plate. Scanning was performed in absorbance mode at 600 nm with winCATS software, using the deuterium light source. The scanning speed was at 20 mm/s, and data resolution was 100 m per step.

### 2.6. Fourier Transform Infrared (FT-IR) Spectroscopy

In the first step, each sample was milled to get fine powder and then mixed with spectroscopic grade KBr powder (1% *w*/*w*) to become a homogenized mixture employing agate mortar and pestle. Second, the mixture was pressed into a pellet. The spectra of samples were acquired by OPUS software subjected to a Bruker vertex-70 instrument. The results were recorded in the mid-IR region (4500-400 cm^−1^) at resolution 4 cm^−1^ and 120 scans [27].

## 3. Results

### 3.1. The Albumin Denaturation Inhibitory Activity

Different organs of the wallflower exhibited high *in vitro* anti-inflammatory activity.  Table 1 represents the percentage inhibition of various concentrations of wallflower extracts along with diclofenac sodium as the standard drug. According to the extract yields of flowers (28.8%), aerial parts (13.8%), roots (6.2%), and seeds (13.36%), the concentration of 1 mg/mL contains equivalent doses of 3.47 mg dried flower, 7.25 mg dried aerial part, 16.13 mg dried root, and 7.49 mg dried seed powders, respectively.

### 3.2. Macroscopic and Microscopic Evaluations

**Figure 2:**
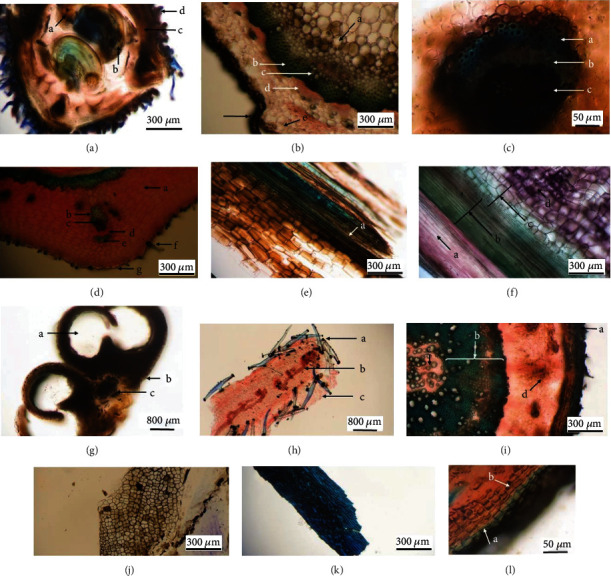
Photomicrographs of transections of different organs of a wallflower. (a) Flower cross-section of the ovary: a: placentation, b: ovule, c: pericarp, d: trichrome. (b) Stem: a: pitch, b: fiber, c: xylem, d: phloem, e: cortex, f: epidermis. (c) Mid-vein of leaf: a: fiber, b: phloem, c: xylem. (d) Fruit: a: parenchyma, b: fiber, c: vessels, d: phloem, e: fiber, f: trichome, g: epidermis. (e) Stem: a: spiral thickening vessel. (f) Stem: a: cortex, b: xylem, c: pitted parenchyma, d: pitch. (g) Cross section of anther: a: pollen sac, b: epidermis, c: connection. (h) Sepal: a: trichome, b: vessels, c: stomata. (i) Root: a: cork, b: secondary xylem, c: primary xylem, d: phloem. (j) Tangential section of seed: inner epidermis. (k) Seed (pitted macrosclereid of the tests). (l) Root bark: a: cork, b: phelloderm.

The transection of the flower ovary showed two ovules, each containing an embryo sac (Figure 2(a)). The transection of anther showed 2-3 cell middle layers that usually disintegrate during anther maturation. The epidermis was observed on the outer layer of anther while the next layer was endothecium, and the inner layer was known as tapetum (Figure 2(g)). Cross-section of sepal represented T shape trichomes and stomata in the epidermis and vessels in the midrib (Figure 2(h)). The fruit consists of three layers; the outermost layer is the exocarp involving the epidermis, the innermost layer is the endocarp which has a fiber layer, and the tissue between them is the endocarp containing parenchyma and collateral vascular bundle (Figure 2(d)). The longitudinal section of the stem showed a vascular bundle consisting of cortex, xylem, and pitted parenchyma, which is the parenchyma inside the pith (Figure 2(f)). The transection of the stem demonstrated an epidermis, cortex, and pith. The stele contains vascular tissue arranged with a collateral bundle around the pith (Figure 2(b)). Also, a collateral vascular bundle in the transverse section of the midrib of the leaf can be observed. The structure of the vascular bundle shows fiber, phloem, and xylem (Figure 2(c)). Furthermore, the results demonstrate it pitted macrosclereid of the tests in seed while testa epidermis involves thickened polygonal cells (1-K). On the other hand, four distinct layers are seen in the cross-section of the root as followed: the outermost layer, which is the root bark. The next layer including primary and secondary phloem, then secondary xylem, and the last layer, in the center, is the primary xylem (Figure 2(i)). The outer epidermal layer, cork, and phelloderm of root bark are shown in Figure 2(l).

### 3.3. Physicochemical Analysis

Physicochemical parameters of major medicinal parts of a wallflower (flower, aerial part, root, and seed) are shown in Table 2.

### 3.4. HPTLC Analysis

**Figure 3:**
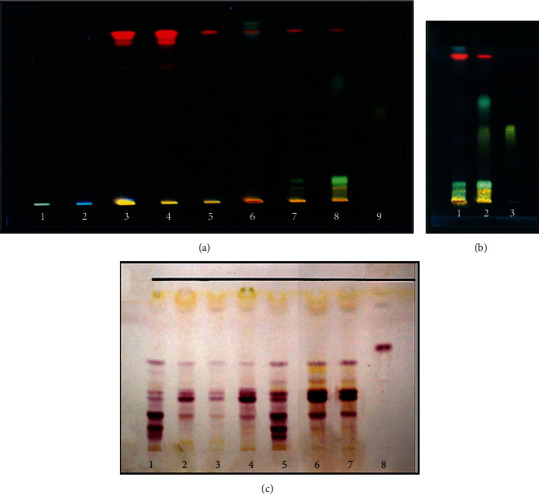
The HPTLC pattern of wallflower organs. (a, b) flavonoids fraction in the methanol extracts: (a) 1: seed, 2: root, 3: fruit, 4: aerial part, 5: sepal, 6: petal, 7: flower, 8: acid hydrolysis of flower, 9: Quercetin; (b) 1: flower, 2: acid hydrolysis of flower, 3: Quercetin; (c) Total cardenolides fraction: 1: seed, 2: root, 3: stem, 4: leaf, 5: fruit, 6: petal, 7: flower, 8: digoxin.

The results of HPTLC analysis of wallflower samples are provided in Figure 3. Flavonoid bands are detected in petals, flowers, and the acid hydrolysed flowers (Figure 3(a)). Our results indicate the presence of at least 4 flavonoid zones with different colors and different *R*_*f*_ values ranging from 0.05 to 0.47 in the acid hydrolysed sample of flowers. The yellow-orange fluorescent zone with the *R*_*f*_ value of 0.47 in the acid hydrolysed sample is found to be quercetin (Figure 3(b)). The profiles of flavonoids are different in sepal and petal samples. Meanwhile, the cardenolide profile of various organs of wallflower is relatively similar, but some differences in the intensity of the HPTLC bands can be seen. The presence of 8 types of cardenolides was observed as eight bands with violet-red zones (vis) and different *R*_*f*_ values ranging from 0.08 to 0.52. Lesser diversity was detected in the stem with 4 sharp zones. Moreover, seed and fruit samples have a higher diversity of cardenolide compounds. Digoxin was tested as a marker in this study (Figure 3(c)).

### 3.5. Fourier Transform Infrared (FT-IR) Spectroscopy

**Figure 4:**
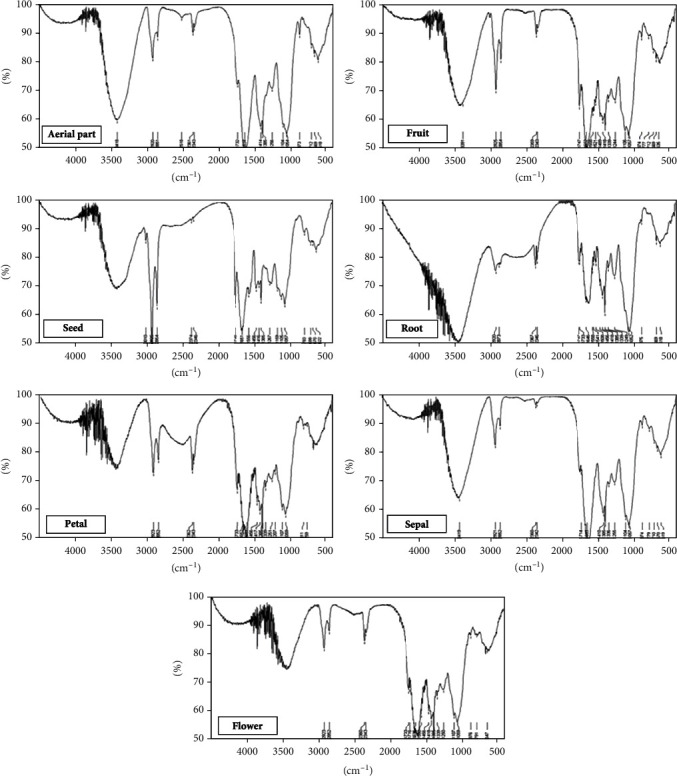
FT-IR patterns of wallflower different organs.

**Figure 5:**
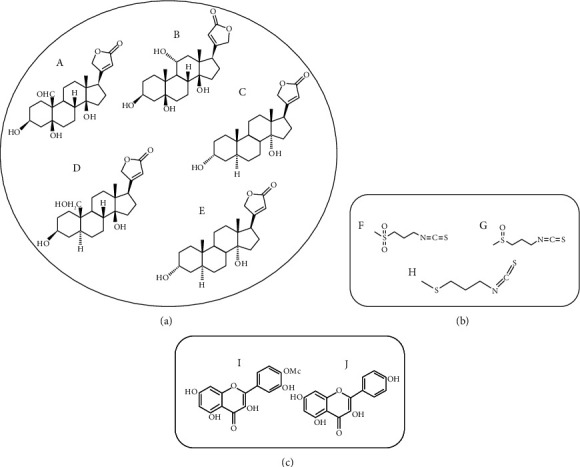
Chemical structure of three essential groups of known compounds in wallflower herb. (a) Cardiac steroids (A: strophanthidin, B: bipindogenin, C: uzarigenin, D: cannogenol, E: digitoxygenin). (b) Isothiocyanates (F: cheiroline, G: iberin, H: ibervirin). (c) Flavonoids (I: isorhamnetin, J: kaempferol).

Infrared spectroscopy as a nondestructive, rapid, simple, and low-cost technique was applied in this study. The general spectra of the aerial part, seed, petal, sepal, flower, fruit, seed, and root of wallflower recorded in the mid-IR region (4500-400 cm^−1^) are demonstrated in Figure 4. Of all known chemical compounds in the wallflower herb [1], approximately three classes of compounds are of medicinal significance (Figure 5). Among these, cardiac steroids, including strophanthidin, bipindogenin, uzarigenin, cannogenol, and digitoxygenin groups, have a complex structure with an aromatic ring. The spectral region at ~3500 cm^−1^ was related to a hydroxyl group (-OH) stretching vibration, at ~2900–2950 cm^−1^ was equivalent to C–H group, at ~2340 cm^−1^ was attributed to isothiocyanate group (–N=C=S), at ~1740 cm^−1^ was due to stretching vibration of the -C=O group, at ~1650 cm^−1^ corresponding to the bending mode of the conjugated C=C of the aromatic ring, and at ~1200-1000 cm-1 was equivalent to the aromatic group structure.

## 4. Discussion

Wallflower (*Erysimum cheiri*) is a popular herb in traditional medicine systems. It has frequently been recommended topically in cerates, lotions, sitz baths, and poultices as an anti-inflammatory drug in a number of ailments such as arthritis, endometriosis, mastitis, and anal fissure by traditional Persian physicians [1, 7, 28]. A literature survey exhibited that only a few studies have been directed on the phytochemical constituents of the wallflower. Also, there is a lack of information about the pharmacological properties of the herb in current medicine. Our recently published study on a traditional formulation containing wallflower extract showed a significant reduction of inflammation scores in anal fissure patients [9]. The present *in vitro* study indicated the solid anti-inflammatory effects of a wallflower compared to diclofenac sodium (see Table 1). These findings are consistent with the anti-inflammatory indications of a wallflower in traditional Persian medicine.

In the next step, we performed different analytical methods, including microscopy, physicochemical, HPTLC, and FT-IR spectroscopy, to determine the quality limits and identification characteristics of wallflower [10, 29, 30]. The highest amount of total ash was determined in the aerial parts (21.52 ± 0.06%). In contrast, the water-soluble ash, acid-insoluble ash, and sulphated ash were higher in seed (34.89 ± 0.26%), root (9.50 ± 0.04%), and aerial parts (28.37 ± 0.57%), respectively. Also, loss on drying amount was ranged from 2.20 ± 0.20% in flowers to 6.00 ± 0.10% in aerial parts. On the other hand, the observed HPTLC fingerprints reflected the complex content and integral characterization of different parts of the wallflower. Flavonoid fingerprinting demonstrated quercetin band in the acid hydrolysed sample of flowers. Cardenolide fingerprinting indicated that all the wallflower organs are rich in cardiotonic steroid compounds. Moreover, the similarity between the cardenolide profile in seeds and other organs of wallflower revealed the presence of the exact identified cardiotonic steroids in seeds as major compounds in other organs. Also, it was found that flowers, fruits, and seeds are the main target organs for cardenolide screening, while flavonoid content is remarkably higher in flowers than in the other organs. On the other hand, according to FT-IR results, the spectra of different parts of the wallflower were rather similar, but some differences in their intensity and shape were observed.

Based on the literature, flavonoids and cardiotonic steroid compounds could be responsible for anti-inflammatory properties. It is noteworthy that cardiac glycosides have shown *in vivo* anti-inflammatory effects in both acute and chronic inflammation models via influence on Na^+^-K^+^-ATPase pump in previous studies [31, 32]. The anti-inflammatory characteristic of cardiac glycosides is not only by addressing the Na^+^-K^+^-ATPase pump but also via other relevant systems such as leukocyte infiltration, fluid extravasation, T_H_17 cell differentiation, immunoglobulin production, cytokine secretion, NFkB activation, cell proliferation, proinflammatory cytokines release, and nuclear receptor ROR*γ*t systems [31, 32]. On the other hand, the flavonoids of wallflower could be responsible for anti-inflammatory activities via different pharmacological mechanisms. For instance, quercetin can interfere with particular biological pathways via reducing the expression of some interleukins such as IL-2 and IL-6, and also iNOS, p38 MAPK, NF-*κ*B, and TNF-*α* levels to induce anti-inflammatory activities [33]. But it is notable that we did not detect any flavonoids in the profile of seed and root samples while they expressed strong anti-inflammatory activities. It could be due to the existence of their cardenolide compounds. In this regard, flower extract has shown the maximum anti-inflammatory activity among the other extracts, maybe because of the synergic effect of flavonoids and cardenolides. Also, based on the traditional Persian medicine implications and recent investigations suggesting anti-inflammatory characteristics for cardenolides, it seems that wallflower topical administration could be considered as a valuable herbal ingredient and natural product for anti-inflammatory purposes in the future. This potential property may be due to the high cardenolide and flavonoid contents of the herb.

## 5. Conclusions

The current study allowed us to describe the histological, phytochemical, and fingerprint features of *E. cheiri*. According to the microscopic results, the wallflower flower (i.e., Erysimi Flores) has anthers with 2-3 cell middle layers that usually disintegrate during maturation. The three layers of the anther are epidermis, endothecium, and the inner one called tapetum (Figure 2(g)). The ovary has two ovules with embryo sacs (Figure 2(a)). Sepals have T shape trichomes and stomata in the epidermis and vessels in the midrib (Figure 2(h)). On the other hand, physicochemical analysis including the total ash, water-soluble ash, acid-insoluble ash, sulphated ash, and loss on drying of the flower was found to be 11.01 ± 0.03%, 5.00 ± 0.03%, 9.23 ± 0.21%, 16.07 ± 0.02%, and 2.20 ± 0.20%, respectively. Furthermore, HPTLC results demonstrated flavonoid bands in petal, flower, and the acid hydrolysed sample of flower (Figure 3(a)). Four different flavonoid zones with different *R*_*f*_ values ranging from 0.05 to 0.47 were detected in acid hydrolysed flowers, and quercetin was represented as a yellow-orange fluorescent band in *R*_*f*_ 0.47 of the acid hydrolysed sample (Figure 3(b)). Flavonoids patterns were different in sepal and petal. Moreover, the cardenolide profile of flowers and different organs of wallflower were rather similar, but some differences in the intensity and diversity of HPTLC bands were observed. On the other hand, the wallflower aerial part (i.e., Erysimi Herba) demonstrates a vascular bundle consisting of cortex, xylem, and pitted parenchyma which are the parenchyma inside the pith (Figure 2(f)). The stele contains a vascular tissue arranged with collateral bundle around pith (Figure 2(b)). Also, collateral vascular bundle in transverse section of midrib of leaf can be observed. The structure of vascular bundle shows fiber, phloem, and xylem (Figure 2(c)). Besides, total ash, water soluble ash, acid insoluble ash, sulphated ash, and loss on drying of aerial part were determined 21.52 ± 0.06%, 7.16 ± 0.06%, 4.05 ± 0.50%, 28.37 ± 0.57%, and 6.00 ± 0.10%, respectively. HPTLC profile of stem demonstrated lesser diversity of cardenolide compounds with 4 sharp zones. On the other hand, wallflower root (i.e., Erysimi Radix) consists of root bark, primary and secondary phloem, secondary xylem, and primary xylem (Figure 2(i)). The outer epidermal layer, cork, and phelloderm of root bark are shown in Figure 2(l). Total ash, water soluble ash, acid insoluble ash, sulphated ash, and loss on drying were determined 5.03 ± 0.03%, 5.04 ± 0.01%, 9.50 ± 0.04%, 21.93 ± 0.11%, and 5.40 ± 0.00%, respectively. Wallflower fruit (i.e., Erysimi Fructus) consists of three layers including exocarp involving epidermis, endocarp composed of a fiber layer, and endocarp containing parenchyma and collateral vascular bundle (Figure 2(d)). HPTLC fingerprint showed a high content of cardenolides in wallflower fruits and seeds. Furthermore, wallflower seed (i.e., Erysimi Semen) contains pitted macrosclereid of the tests while testa epidermis showed thickened polygonall cells (Figure 2(k)). Total ash, water soluble ash, acid insoluble ash, sulphated ash, and loss on drying were determined 6.95 ± 0.06%, 34.89 ± 0.26%, 1.51 ± 0.01%, 7.03 ± 0.03%, and 4.35 ± 0.05%, respectively. The results of this study could provide a basis for discovering adulterations and establishing a pharmacopoeial monograph to be served as identifiers of different organs of *E. cheiri*.

## Figures and Tables

**Table 1:** Albumin antidenaturation activity of wallflower and diclofenac sodium.

Concentration [mg/mL]	% inhibition (mean ± SD) (*n* = 3)				
	Flower extract	Aerial part extract	Root extract	Seed extract	Diclofenac sodium
1.25	12.88 ± 1.31^a^	12.12 ± 4.73^a^	15.15 ± 4.2^a^	6.06 ± 8.60^e^	0.76 ± 2.62^f^
2.5	20.45 ± 2.27^b^	16.16 ± 5.25^b^	21.97 ± 3.47^b^	12.12 ± 2.62^a^	3.79 ± 1.31^e^
5	43.18 ± 4.55^c^	31.06 ± 5.72^b^	35.61 ± 1.31^c^	19.7 ± 1.31^b^	9.85 ± 1.31^a^
10	60.61 ± 11.21^d^	50.76 ± 1.31^d^	64.39 ± 6.56^d^	24.24 ± 3.47^b^	21.97 ± 3.47^b^

Data are given as mean ± SEM (*n* = 3). Data sets with different alphabetical superscripts are significantly different (*p* < 0.05).

**Table 2:** Physicochemical evaluation of different parts of *E. cheiri* (*n* = 3).

Particulars	(% *w*/*w* ± SD)			
	Flower	Aerial part	Root	Seed
Total ash	11.01 ± 0.03%	21.52 ± 0.06%	5.03 ± 0.03%	6.95 ± 0.06%
Water soluble ash	5.00 ± 0.03%	7.16 ± 0.06%	5.04 ± 0.01%	34.89 ± 0.26%
Acid-insoluble ash	9.23 ± 0.21%	4.05 ± 0.50%	9.50 ± 0.04%	1.51 ± 0.01%
Sulphated ash	16.07 ± 0.02%	28.37 ± 0.57%	21.93 ± 0.11%	7.03 ± 0.03%
Loss on drying	2.20 ± 0.20%	6.00 ± 0.10%	5.40 ± 0.00%	4.35 ± 0.05%

## Data Availability

All the data used to support the findings of this study are included within the article.
